# Advances in radiotherapy enhancing the efficacy of immune checkpoint inhibitors in malignant

**DOI:** 10.3389/fonc.2025.1611036

**Published:** 2025-07-01

**Authors:** Yao Liao, Jinjing Deng, Xiyue Yang, Decai Wang, Xiaobo Du

**Affiliations:** ^1^ Sichuan Clinical Research Center for Radiation and Therapy, Mianyang Central Hospital, School of Medicine, University of Electronic Science and Technology of China, Mianyang, Sichuan, China; ^2^ Department of Oncology, Mianyang Central Hospital, School of Medicine, University of Electronic Science and Technology of China, Mianyang, Sichuan, China; ^3^ Department of Orthopedics, Sichuan Science City Hospital, Mianyang, Sichuan, China; ^4^ Department of Urology, Mianyang Central Hospital, School of Medicine, University of Electronic Science and Technology of China, Mianyang, Sichuan, China

**Keywords:** malignant tumors, immune checkpoint inhibitors, radiotherapy, tumor therapy, cold tumors

## Abstract

The introduction of immune checkpoint inhibitors (ICIs) has facilitated the elucidation of the mechanisms underlying the remote effects observed in tumor therapy and has demonstrated significant promise for treating several advanced tumors. However, the natural resistance of “cold tumors” remains a challenge that ICIs alone cannot overcome. Radiotherapy (RT) has been shown to enhance anti-tumor immunity by generating *in situ* antigens or antigenic fragments derived from tumor cells and local immune cell DNA, thereby attracting more immune-presenting cells to the tumor site. This process promotes the conversion of immune cells into anti-tumor effector cells and enhances the efficacy of ICIs, most likely reflecting the mechanism of the abscopal effect (ABE). Alternatively, ABE achieves optimal efficacy when anti-tumor effects synergistically enhance systemic immunity. This review delineates the molecular mechanisms underlying the distant compartment effect and summarizes clinical studies on enhancing immune checkpoint inhibitors through various RT techniques.

## 1 Introduction

Immune checkpoint inhibitors (ICIs) have transformed the treatment landscape for malignant tumors, becoming integral to many first-, second-, and subsequent-line therapies for advanced cancers ICIs. However, some tumors were inherently immunologically “cold” and thus unresponsive to ICIs. Moreover, tumor heterogeneity could lead to adaptive resistance in initially ICI-sensitive tumors. Systemic administration of ICIs combined with other treatment, particularly local therapy, has emerged as a optimal strategy to overcome both primary and acquired resistance to ICIs in clinical practice.

RT, the most widely used local treatment for advanced malignant tumors patients (1, 2), could trigger abscopal effects and improve the efficacy of ICIs by changing the local and systemic immune microenvironment. In 1953, Mole firstly reported the phenomenon of distant unirradiated tumors regressing following RT of a single tumor lesion, which he termed the abscopal effect (ABE) (3). In the subsequent decades, ABE was rarly reported and seemed as occasional and unintentiona. However, in 2012, Postow reported a case of ABE induced by ipilimumab and RT for melanoma which revealed the potential of combining RT with ICIs to increase the likelihood of triggering ABE (4).Since then, RT plus ICIs strategys have attracted attention of clinical and preclinical research, owing to its ability to control systemic tumors through localized treatment and reducing the risk of metastasis (5), in brief, its potential to induce ABE.

Over the past two decades, accumulating preclinical and clinical evidence has demonstrated that various RT techniques could reshape the immune microenvironment, thereby enhancing the therapeutic efficacy of ICIs.The combination of stereotactic body radiotherapy (SBRT) with immune checkpoint inhibitors (ICIs) acts as an immunotherapeutic catalyst by generating *in situ* tumor antigens, which subsequently activate and recruit antigen-presenting cells (APCs), thereby eliciting robust local and systemic anti-tumor immune responses (6, 7).Low-dose radiotherapy (LDRT) has been demonstrated to remodel the immunosuppressive tumor microenvironment, effectively converting immune “deserts” into immunologically active niches through the phenotypic reprogramming of APCs into anti-tumor effectors (7, 8).Spatially fractionated radiotherapy (SFRT) enhances immune responses by leveraging the distinct immunomodulatory effects of high-dose and low-dose radiation regions within the tumor (9).Emerging ultra-high-dose-rate radiotherapy (FLASH-RT) not only achieves superior tumor control but also significantly reduces normal tissue toxicity, offering a promising approach for immunomodulation (10).Furthermore, novel combinatorial strategies—such as LDRT plus SBRT or SFRT combined with FLASH-RT—have demonstrated synergistic efficacy, suggesting unexplored mechanistic insights and therapeutic potential for radio-immunotherapy (11–14).

This review discusses preclinical and clinical studies on RT combined with ICIs to enhance treatment efficacy and trigger ABE.

## 2 Molecular mechanism underlying ABE

The mechanism underlying ABE remains under investigation; however, current research suggests that ABE primarily involves a T cell-mediated systemic immune process that reduces the risk of distant metastasis and helps to treat distant metastasized tumors in conjunction with local therapy (15).

### 2.1 RT generates *in situ* antigens, activates antigen-presenting cells, and alters the body’s immune environment to induce ABE

RT induces irreversible DNA damage within the target area, with broken DNA fragments exposed in the cytoplasm. These ectopic DNA fragments activate immune-presenting cells (APCs), prompting the maturation and proliferation of dendritic cells (DCs) (16). The immune system recognizes pathogens and damaged cells through APC by recognizing damage-associated molecular patterns (DAMPs) and pathogen-associated molecular patterns (PAMPs), ultimately inducing immune responses (17). The most common DAMP is high-mobility group box 1 (HMGB1), followed by heat shock proteins, reticulin, and S100 acidic calcium-binding protein (18, 19). These DAMPs are captured by APC and presented via the major histocompatibility complex (MHC), which activates CD8^+^ T cells or CD4^+^ T lymphocytes. For example, HSP70 binds to toll-like receptor 4 and CD91, which in turn activates CD8^+^ somatic and NK cells (20), and, unlike T cells, activated NK cells do not require antigenic sensitization but instead directly recognize and kill tumor cells. Furthermore, the oxidized DNA from the mitochondria or nuclei exposed to the cytoplasm following RT can bind to Toll-like receptor 9, which induces an inflammatory response (21).

Moreover, activation of CD8^+^ T cells induces the release of various cytokine, such as tissue necrotic factor (TNF)-α and interferon (IFN)-γ, creating a positive feedback loop that further activates CD8^+^ T cells (22) and allows M2-type polarized macrophages to convert into M1-type and exert anti-tumor immune effects (23). Beyond enhancing antitumor immune cell activity, radiotherapy concomitantly suppresses immunosuppressive cell populations, synergistically augmenting systemic antitumor efficacy. RT has also been shown to upregulate PD-1 expression by Tregs (24–26). These cytokines stimulate anti-tumor immunity in tumors located elsewhere in the body when they travel through the blood and lymph fluid to sites distant from the RT location, thereby inducing ABE. Yang et al. detected elevated levels of serum IFN-γ following RT for localized tumors, which promoted tumor regression in the radiation field (27). Although some previous studies focused on RT have suggested that some cytokines may bimodal and have paradoxicalimmunosuppressive attributes as well, in the era of radotherapy plus ICIs, more studies support the enhancement of its anti-tumor activity (17, 18).

### 2.2 cGAS-STING is an essential pathway in the abscopal effect

Several molecular signaling pathways involved in ABE have been identified. Of these, accumulating evidence highlights the pivotal role of cyclic guanine nucleotide-adenine nucleotide synthetase-interferon gene-stimulating factor (cGAS-STING) pathway.Notably, the cGAS-STING pathway not only directly activates antitumor immune cell-mediated cytotoxicity, but also initiates multiple downstream signaling cascades including (1): PD-L1/JAK-STAT (2), NF-κB (3), RAS-ERK1/2, and (4) cGAS-STING-IFN axes. These interconnected networks collectively potentiate antitumor immunity. Importantly, therapeutic modulation of these pathways may induce adenine base editing (ABE) effects. DNA fragments generated by RT infiltrating the cytoplasm and bind to cyclic guanosine-phosphate adenosine monophosphate (cGAMP) synthase (cGAS) proteins, increasing the levels of intracellular cGAMP. Intracellular cGAMP binds to interferon-gene-stimulating (STING) proteins, which induce the interaction between IFN-1 (IFN-alpha and IFN-beta) and IFNAR1/2 receptors. Furthermore, IFNAR1/2 receptors directly activate NK cells and macrophages, promoting DC maturation and proliferation and activating CD4^+^ T lymphocytes and CD8^+^ T cells (28–31).

However, the cGAS-STING pathway can potentially promote tumor cell proliferation while playing the role of a “guardian of justice.” The cGAS-STING pathway is involved in the NF-κB-induced elevation of interleukin (IL)-6 expression, which can inhibit tumor and oxidative stress and promote tumor proliferation (32). Sumaiah et al. reported that the non-classical STING pathway elevates IL-6 in caffeine-damaged tumor cell DNA. Additionally, the authors demonstrated that blocking this non-classical pathway or inhibiting the expression of downstream extracellular signal-regulated kinases 1 and 2 led to a reduction in IL-6 levels. This finding suggests a potential means to counteract the tumor growth-promoting effect of comedones (33). Meanwhile, the immunosuppressive activity of cells generated by apoptotic cells early in RT can be mitigated by raising the dose in a single session and reducing the overall number of sessions (34). Moreover, cGAS-STING upregulates programmed death-ligand (PD-L) 1 by activating the JAK-STAT pathway; this enhances the systemic efficacy of ICIs (35). Wang et al. discovered that blocking cGAS-STING expression in a mouse melanoma model diminished the antitumor efficacy of PD-L11 inhibitors. In contrast, intramuscularly injecting cGMP into mice significantly enhanced the antitumor effects of PD-L1 inhibitors, suggesting that the cGAS pathway can initiate T cell antitumor immunity via anti-PD-L1 (36). Notably, the gene for the DNA 3′-5′ nucleic acid exonuclease DNase III (Trex1) is one of the most important negative regulators of cGAS. Vanpouille-Box et al. found that Trex1 could degrade broken DNA in the cytoplasm following radiation therapy, preventing cGAS activation. This weakening of tumor immunogenicity was more pronounced when a single RT dose exceeded 10–12 Gy.

Moreover, ABE has been observed in the locally irradiated group with knocked down Trex1 expression followed by treatment with a cytotoxic T-lymphocyte-associated protein (CTLA)-4 inhibitor in a mouse model of bilaterally inoculated tumors (37, 38).

In summary, local RT can achieve results akin to a catalyst through T-cell-mediated immunity, with ABE potentially representing one of the best outcomes of the “catalyst” (39). Hence, determining how RT induces more frequent and stronger ABE is a prominent area of research.

## 3 Rationale for the combination of RT and immunotherapy

ICIs have become pivotal agents in the systemic management of advanced malignancies. However, acquired resistance frequently emerges during ICI therapy. Then combinatorial approaches incorporating local treatments are often employed clincally (40). RT was the most widely utilized local treatment (1, 2),because it could induce rapid tumor cell death within hours, generate neo-antigens *in situ*, enhance the immunogenicity of tumor cells hereupon and facilitate improved T-cell recognition. Furthermore, RT remodels the tumor microenvironment (TME), promoting enhanced infiltration of immune cells (41–43) and activating the previously mentioned abscopal cGAS-STING signaling pathway (25, 44, 45). Notably, RT upregulates critical immunomodulatory molecules on immune cells, including ICAM-1, Fas, and MHC class I (46–48). This upregulation potentiates a rapid augmentation of systemic anti-tumor immunity. Conversely, downregulation of these molecules is associated with immune tolerance and evasion. Importantly, these immunostimulatory effects exhibit a dose-dependent relationship within the radiation dose range of 1 to 20 Gy (49).

## 4 Different RT parameters enhance the efficacy of immune checkpoint inhibitors

The total and fractionated doses of RT, different drugs for ICIs, and sequence of RT and ICIs influence the efficacy of the combination regimen.

### 4.1 Neither conventional segmentation nor a single high dose of RT is optimal.

Okuma et al. reported a case of hepatocellular carcinoma with lung and mediastinal lymph node metastases. the lung tumor outside the radiation field also reduced significantly after administering 60.75Gy/27F to the mediastinal lymph nodes (50). However, ABE triggered by conventional segmentation has rarely been reported, possibly because the killing of lymphocytes by conventional segmentation is much greater than that of SBRT (51). Furthermore, a study comparing the changes in the vasculature when 60 Gy was divided into three, five, and eight irradiations reported a reduction in the collapse of the vasculature and unfavorable migration of T cells immediately after a single 60 Gy/3F RT (52). Additionally, Song et al. reported destruction of the vasculature and hypoxia, among other consequences, after 20 Gy RT; hence, it was unfavorable to the production of ABE (53).

### 4.2 SBRT is one of the most effective RT techniques for inducing ABE

SBRT is defined as a dose of ≥ 5 Gy per fraction. Many split doses can induce ABE, with 24GY/3F showing the most evidence.

Preclinical studies have confirmed the immune-enhancing efficacy of SBRT regimens, with a total dose of 15–30 Gy divided into 2–5 doses. In a mouse non-small cell lung cancer model, better tumor control was found in 15 Gy/1F group than 15 Gy/5F group,through stronger T-cell activation and infiltration (54). Schaue et al. compared the greatest reduction in melanoma tumor volume at the same total 15Gy, 7.5 Gy/2F showed the best tumor control and tumor immunity while maintaining low Treg numbers (55). Furthermore, Dewan et al. used a different SBRT segmentation method combined with CTLA-4 inhibitors in a mouse breast cancer model and better local control and ABE efficacy was found with 24 Gy/3F than with 20 Gy alone and 30 Gy/5F. The efficacy of RT is proportional to the activation and proliferation of CD8^+^ T-cells in peripheral blood (56). Vanpouille-Box et al. combined CTLA-4 with 24 Gy/3F SBRT to induce ABE in homozygous mice and found that a split dose of 4–12 Gy upregulated IFN-β production and secretion through the cGAS-STING pathway. The latter increased tumor efficacy against ICIs, whereas a single 20 or 30 Gy of RT-induced Trex1-mediated degradation of cytoplasmic DNA and abrogated the effects of IFN-β (37). Moreover, Mihaylov et al. at the Miami Myrtle School of Medicine combined RT and ICIs in mice with a high percentage of ABE, approximately 27% (4/15) (57). Melanoma mouse models were treated with 3× 9.18 Gy for 1 week or 5×6.43 Gy for 10 days, with anti-PD1 antibodies administered weekly. Both long- and short-course RT combined with PD1 inhibitors triggered local and systemic antitumor efficacy via CD8^+^ T cells. The tumor control was also similar in the RT field for both radiotherapies (58). The ABE of varying divided radiotherapies mapped in a mouse model of colon cancer showed that the 24 Gy/3F modality had the strongest systemic immune antitumor effect, either alone or in combination with a PD1 inhibitor (59). Animal studies by Mortezaee et al. confirmed a stronger antitumor immune efficacy of 8–12 Gy than lower doses (60).

In the following few decades of ABE’s discovery, clincal studies on RT-induced ABE were mostly case reports, and the data provided were heterogeneous,which implied that identifying the patterns of ABE through summarizing the literature posed a significant challenge. Reynders et al. analyzed 23 case reports with a median age of 64.5 years and single doses ranging from 1.2 to 26 Gy with a mean total dose of 32 Gy (12–60.75 Gy). Regarding the site of RT, 8 patients were treated with RT for the primary tumor and 15 with RT for the metastatic site. ABE occurred 1–24 months post-RT. Furthermore, the median ABE duration was 5 months, with a median follow-up of 13 months (61). An analysis by Brix showed that ABE was more common in advanced or older progressive and metastatic melanomas, lymphomas, renal cell carcinomas, and hepatocellular carcinomas in patients who received palliative RT (62).

The safety of RT combined with ICIs in patients was first validated by Brahmer in 2012, followed by several other clinical studies (63). A phase I clinical trial evaluated the efficacy of pabolizumab combined with SBRT in 24 patients with metastatic cancer, of which 12 received 24 Gy/3F, and the other 12 received 17 Gy/1F. Two patients in the 24 Gy/3F group who had previously progressed on PD-1 inhibitor therapy were included, and the combination was effective, with the efficacy persisting for 9.2 and 28.1 months. One patient in the 17 Gy/1F group experienced complete remission, while two patients continued to have stable disease (7.4 and 7.0 months, respectively). Further analysis showed that the combination therapy induced PD-1 expression and increased CD8^+^ T cell count (64). Additionally, a secondary analysis of KETNOTE001 showed that progression-free survival (PFS) could be prolonged from 2.1 to 4.4 months and overall survival (OS) was also prolonged from 5.3 to 10.7 months if SBRT was received before pabolizumab treatment in advanced non-small-cell lung cancer. The efficacy was even more pronounced if the site of RT was extracranial. Chen et al. conducted two retrospective studies of SBRT combined with ICIs in metastatic non-small cell lung cancer, 17 of 33 patients were in the SBRT+CTLA4 inhibitor group, while 16 were in the SBRT+PD-1 inhibitor group. The field response rates of the two groups were 24% vs. 37%, and the systemic metastatic lesion efficiencies were 24% vs. 56%. The PFS rates at 3, 6, 12, and 18 months were 76% vs. 94%, 52% vs. 87%, 31% vs. 80%, and 23% vs. 63%, respectively (p = 0.02), and the OS rates at 6, 1, and 18 months were 76% vs. 87%, 47% vs. 80%, and 39% vs. 66%, respectively (p = 0.08). This suggested that SBRT combined with different ICIs to manage the same tumor type had varying immune-enhancing efficacies, which could result in different survival benefits for patients. Additionally, PD-1 inhibitors combined with SBRT had an enhanced ability to induce ABE with longer-lasting efficacy (65). Almost all current ABE case reports are peripheral. However, Lin et al. reported a case of ABE observed extracranially with second-line atelizumab following SBRT for brain metastases from lung cancer. The authors suggested that immune cells could cross the blood-brain barrier following RT, altering the immune microenvironment of head tumors. However, further studies are required to clarify this mechanism (66).

SBRT-based partial Tumor irradiation (SBRT-PATHY) is a novel RT which aimed to induce ABE by targeting hypoxic clonogenic cells by sparing the peritumoral immune microenvironment and regional circulating lymphocytes, has shown the potential to induce ABE either alone or in combination with ICIs. SBRT-PATHY has been developed by Tubin et al, to enhance the radiotherapy therapeutic ratio of advanced lung cancer (67, 68). At present, researches of SBRT-PATHY mainly focuses on patients with bulky tumors, and have already been confirmed in both clinical retrospective studies and prospective phase II clinical studies that it did induce ABE and enhance the anti-tumor efficacy (69–71). However, we believe the most remarkable aspect of SBRT-PATHY is not the above-mentioned therapeutic effect but its systemic anti-tumor efficacy and reversal of ICIs resistance shown by only SBRT-PATHY therapy which may be the most promising value of this technology. It may even be expected to further reveal the potential mechanism of ABE.

Personalized ultrafractionated stereotactic adaptive radiation therapy (PULSAR) is an novel RT approach explored at the University of Texas Southwestern Medical Center for managing brain metastases (72, 73). It tries to evaluate tumor changes during treatment to personalize and adjust radiation delivery, aiming to optimize therapeutic outcomes while minimizing side effects. PULSAR is designed to deliver high-dose radiation at 2 to 4-week intervals, about 20-30GY/4-6F. It has been reported several times in preclinical and small-sample clinical studies, shows PULSAR plus ICIs was more complementary than traditional daily fractions plus ICIs in this preclinical model and better promise for optimizing patient management and reducing the risks of treatment (74–77). The fractionation doses of PULSAR are consistent with conventional SBRT, primary distinctions lying in treatment intervals and adaptive planning implementation. Emerging PULSAR studies demonstrate that radiotherapy-sequenced ICIs administration (RT→ICIs) potentiates systemic therapeutic efficacy more effectively than concurrent approaches which was align with established principles of immunologic cascade activation of antitumor *in vivo*.

However, SBRT has several drawbacks. First, its bioequivalent dose is often high, making it challenging to receive secondary RT following uncontrolled or recurrent treatment at the same or adjacent sites. Second, SBRT cannot be planned for multiple sites of advanced tumors at the same isocenter, making accurate dose calculations for the target area and organs at risk difficult. However, progress in LDRT, which modifies the tumor immune microenvironment, has overcome SBRT’s limitations.

### 4.3 LDRT holds new promise for modulating the immune microenvironment in localized therapy

The Perez and Brady Principles and Practice of Radiation Oncology divides RT by fractionated dose: LDRT refers to doses of 0.1–1 Gy per session; high-dose RT refers to doses of ≥ 8 Gy per session; conventional fractionation refers to doses of 1.8–2.2 Gy per session (78).

LDRT reshapes the tumor’s immune microenvironment without damaging lymphocytes. Notably, inadequate anti-tumor T-cell migration is the primary reason for the poor efficacy of ICIs. LDRT activates immunity through the TH1 subpopulation of CD4^+^ T-cells and promotes proliferative activation of primitive T-cells (79). In a pancreatic cancer model, Klug et al. observed that LDRT enhanced the proliferation of tumor-specific T cells by inducing endothelial cell activation and the expression of TH1 chemokines and normalized the abnormal vasculature of the tumor. This promotes the recruitment of tumor-specific T cells and M1 polarization of macrophages to exert antitumor effects (80). Classical radiobiological research has shown that a 10% lethal dose for CD4^+^ T cells and CD8^+^ T cells ranges from 3.32 to 3.84 Gy (81). Thus LDRT reshapes the tumor microenvironment without killing T lymphocytes of the radiation field and flowing bloodstream. On the contrary, LDRT can promote the homing and activation of T lymphocytes, as well as their tumor infiltration and cytotoxic effects. Compared with doses >1 Gy, 0.5 Gy LDRT induces enhanced T cell infiltration. It also facilitates the polarization shift of M2-type macrophages toward the M1-type, thereby synergizing with CD8^+^ T cells to exert antitumor effects. These immunomodulatory changes in the tumor microenvironment resemble the therapeutic outcomes achieved through pharmaceutical interventions promoting dendritic cell (DC) maturation and proliferation (82). Herrera et al. validated in both animal models and phase I clinical trials across multiple cancer types (including ovarian and lung cancers) that LDRT can remodel the immune-desert tumor microenvironment by enhancing T lymphocyte infiltration through both naive CD4+ T cells and adaptive CD4+ T cells in the immune microenvironment, achieving NKG2D-dependent tumor control (13).

LDRT can sensitize chemotherapy and reverse chemoresistance. Ngoi et al. conducted a phase I clinical trial of whole abdomen LDRT combined with paclitaxel weekly in 10 platinum-resistant ovarian cancer patients, 9/10 achieved biochemical efficacy (> 50% decrease in CA125) (83). Saikat Das et al. conducted a phase II single-arm trial to evaluate the efficacy of LDRT combined with a paclitaxel+carboplatin regimen as neoadjuvant treatment for IIB-IIIB cervical cancer prior to radical RT and showed an overall response rate of up to 100% in 24 patients (84). Studies on LDRT for other tumor types have also been conducted (85, 86). In a phase II-III clinical trial, the combination of LDRT with induction chemotherapy in locally advanced nasopharyngeal carcinoma and no 5-year survival benefits was reported; The author conjectured that chemotherapy was not the best option for the LDRT combination (87). Current evidence suggests, however, that the result is more likely attributable to complete disruption of cervical lymphatic drainage due to the high-dose radiotherapy administered for nasopharyngeal carcinoma subsequently.

LDRT can improve the efficacy of ICIs through various combinations. There are some key preclinical studies on the combination of LDRT and SBRT. Liu et al. performed whole-body 0.1 Gy LDRT on mice initially using colon and breast cancer models, and subsequently, administering 24Gy/3F RT to the primary tumors 3 days later. It was found that the growth of primary and metastatic tumors was reduced, with increased CD8^+^ T-cell infiltration in metastatic sites, decreased MDSCs and M2-polarized macrophages, and suppressed metastatic ability (88). Savage et al. administrated 22 Gy/1F followed by 0.5 Gy/4F/2 days of LDRT to the same tumor in mouse models of lung and breast cancer, found this new approach could delay the tumor growth in mice, prolong survival, increase immunosuppression, reduce immunosuppressive Treg cells and M2 polarized islets, and increased T-cell infiltration were found (89). Barsoumian demonstrated the potent immunomodulatory effect role of LDRT in improving the immune microenvironment and enhancing the therapeutic efficacy of several different ICIs in lung adenocarcinoma (89–91). Monjazeb et al. compared the efficacy of ICIs combined with LDRT or SBRT in the multiline treatment of microsatellite-stabilized metastatic colorectal cancer after progression. The LDRT group received 2 Gy/4F for 2 days before the first 2 cycles of 1–4 cycles of ICIs, which was administered twice a day for a total of 4 cycles. Meanwhile, the SBRT group received 24 Gy/3F for 24 days within the first cycle and 24 Gy/3F for 3 days after the second cycle; the 24 Gy/3F was split within the first cycle. However, no systemic antitumor response was induced in either the SBRT or LDFRT groups combined with PD-L1/CTLA-4 inhibitors. Tumor micronuclei/primary nuclear rupture was observed in two patients in the LDRT group, and nuclear rupture was associated with activation of the GAS/STING pathway. These findings provide a rationale for exploring more optimized LDRT parameters (92). Furthermore, Patel et al. compared the efficacy of metastatic lesion SBRT+/-LDRT in 74 patients with lung cancer and melanoma that progressed on ICIs with the continuation of immunotherapy. Follow-up was 13.6 months, with 39 enrolled in the SBRT group and 35 in the SBRT+LDRT group. The 4-month disease control rate in the SBRT+LDRT group was 37% vs. 47%, the objective efficacy rate was 26% vs. 19%, and the radiation field control rate was 23% vs. 11% (93). The immunosuppressive environment of the liver leads to poor efficacy of various systemic therapies after hepatic metastasis of tumors (94). A phase I clinical trial showed that whole or partial liver RT in patients with liver metastases could altered the poor prognosis of liver metastases. In addition, the preliminary results of the corresponding phase II trial suggest that the local control rate of liver metastases vs. lung metastatic lesions was 71% vs. 29%, respectively, emphasizing a final result for these promising outcomes (11).

Overall, LDRT demonstrated potent yet low-toxicity efficacy in remodeling the immunosuppressive tumor microenvironment, particularly in reversing immune-desert phenotypes. Its activity in chemotherapy- and ICI-resistant populations warrants further translational and clinical investigation.

### 4.4 Spatially fractionated radiotherapies

SFRT, also known as grid RT, has been used to deliver a high but uniform dose of radiation to tumors by placing a regular mesh of lead plates in the path of the radiation after it leaves the collimator and before it enters the body, which blocks the beam in nonporous areas. This therapy is divided into four main subtypes based on the development of RT: two-dimensional grid-like technology (GRID RT GRID-RT), three-dimensional lattice RT (LRT), small-beam RT (minibeam RT MRT), and microbeam RT (MBRT). The four subtypes differ primarily in the beam size. Notably, the dose to the unobstructed area is the peak dose, while the one to the obstructed area is the valley dose, used in the early stages of large tumors to control the tumor while reducing the RT dose to the surrounding normal tissues.

Preclinical studies have shown that SFRT alters the immune microenvironment and induces ABE. In the B16F10 animal model, Bazyar et al. found that combining an immune detection inhibitor with MRT increased the efficacy of immune checkpoint inhibitors. In contrast, the anti-tumor effect of this combination disappeared with the addition of an inhibitor of CD8a or in T-cell immune-deficient Rag-/- mice, suggesting that SFRT enhances the efficacy of immune checkpoint inhibitors via CD8^+^ T lymphocytes (95). Bertho et al. used a glioma rat model to compare normal and immunodeficient rats receiving 30Gy of MBRT and the immune cells in the tumors of the two groups with those in the peripheral blood. The authors found that MBRT induced both T and B lymphocyte infiltration. However, mice with completely regressed tumors following RT could not be inoculated with these tumor cells again, confirming that high-dose RT with MBRT mediates anti-tumor immunity via T lymphocytes and produces a durable immune response (96). In addition, differences in T lymphocyte-mediated efficacy have been observed in several other animal models, including mouse lung cancer and mouse triple-negative breast cancer models of SFRT (97). These studies and others have highlighted the changes in the levels of the inflammatory factors IL-10, IL-4, IL-6, TNF-α, and IFN-γ secreted by lymphocytes after tumors treated with SFRT, as well as the changes in the chemokines IL-2 and CXCL1 (98). A single SFRT treatment also showed antitumor efficacy against unirradiated tumors in some bilateral tumor models (97, 99, 100), suggesting that combining SFRT and immune checkpoint inhibitors is promising to improve antitumor efficacy.

In the era of ICIs, SFRT has beginning to show clinical promise. Despite its century-long history, reports on the immunomodulatory effects of SFRT remain scarce. Early clinical studies primarily focused on safety and toxicity, though some evaluated immune responses. For instance, Sathishkumar et al. demonstrated that GRID-RT (a form of SFRT) significantly elevated TNF-α levels in peripheral blood at three post-treatment time points in 34 patients, correlating with improved tumor control in 2002 (101). Notably, case reports highlight SFRT’s potential to enhance immune checkpoint inhibitor (ICI) efficacy. In 2021, Jiang et al. described an advanced NSCLC patient with rapid progression who achieved complete remission after combining LRT, conventional RT, SBRT, and ICI (102). Similarly, Massaccesi et al. reported a case of renal cell carcinoma that progressed on third-line immunotherapy following progression on targeted therapy. Partial tumor remission was achieved using a combination of SFRT and ICIs.These findings suggest that SFRT may overcome ICI resistance, particularly in bulky tumors.

Although high-quality evidence remains limited, tumor immunology experts hypothesize that SFRT remodels the immunosuppressive tumor microenvironment, with preclinical data implicating the low-dose tumor periphery as a critical zone for immune potentiation. Further mechanistic and clinical validation is warranted

### 4.5 FLASH-RT

The concept of FLASH-RT was proposed by Dewey and Boain in 1959; however, it did not gain attention in the field of oncology until 2014 (103, 104). FLASH-RT, characterized by an ultra-high dose rate of ≥ 40 Gy/s and a RT delivery time of < 200 ms, has become important in the field of RT since it demonstrates different degrees of normal tissue protection while controlling tumors. There are different degrees of protection of normal tissue during RT (105, 106).

The immunomodulatory effects of FLASH-RT have been observed in some preclinical studies. Simmons et al. found that cytokines, such as IL-6, IL-1β, TNF-α, KC/GRO, and IL-4, were elevated in the hippocampus after conventional RT in their study on cognitive function following whole-brain RT in mice, but FLASH-RT showed only three cytokines (IL-1b, TNF-α, and KC/GRO) evaluate and the elevations were much lower, suggesting that FLASH-RT attenuates radiculitis and reduces cognitive impairment following RT. Some of these cytokines are involved in the immune response (107). A comparison of conventional RT with FLASH-RT in the abdominopelvic cavity in an ovarian cancer model revealed that FLASH-RT irradiation increased the infiltration of T cells within the tumor at earlier time points compared with conventional irradiation. Furthermore, FLASH-RT irradiation increased the number of cytolytic CD8^+^ T cells at later time points and enhanced the efficacy of PD-1 therapy (108). In contrast, increased levels of CD3 tumor-infiltrating lymphocytes and decreased levels of regulatory T cells in ovarian cancer correspond to improved prognosis and survival (109). In a mouse model of intestinal cancer, Shi et al. found that FLASH-RT attenuated radiation enteritis by reducing CD8+ T cell-mediated deleterious immune responses when combined with ICIs or in mice with knockout of PDL-1, compared with conventional RT. This study also provided a theoretical basis for using FLASH-RT in combination with ICI (10).

Overall, as stated in a 2024 review (110), FLASH-RT is a novel RT modality, and reports on its immune microenvironmental aspects are limited. Electron beams, X-rays, and protons can produce FLASH-RT effects at ultra-high doses. However, The specificity of FLASH-RT for the immune microenvironment, its combination methods with ICIs, its integration with traditional radiotherapies, and the theoretical mechanisms explaining its distinctions between tumor and normal tissues remain underexplored in preclinical and clinical studies.

### 4.6 The sequence of RT and ICIs is another hot topic of current research.

*In vivo*, eliciting robust antitumor responses requires the initiation and localized amplification of a cascading immune reaction. The sequential engagement of immune components is essential to induce positive feedback within this cascade. To achieve systemic ABE effects, specific molecular ‘switches’ must be activated in accordance with fundamental cascade principles, thereby propagating the reaction throughout the organism. thus the temporal coordination between RT and ICIs emerged as a critical determinant in ABE research (111).

Considering the theory that RT provides *in situ* antigens for immunization, several studies have used RT followed by combined ICIs, and better efficacy observed (75, 76, 112). Secondary analysis of KENOTE-001 showed patients received RT before pabrolizumab had significantly benefited in PFS (6.3 vs. 2 months) and OS (5.3 vs. 11.6 months) (112). Furthermore, the classic PACIFIC study showed that immunomaintenance therapy after concurrent RT in stage III could provide patients with a PFS benefit of approximately 1 year, with a 4-year OS rate of 36.3%–49.6% (113).

### 4.7 Multisite radiotherapy can enhances ICIs efficacy and trigger ABE

Generating more *in situ* antigens via RT at more sites has also produced satisfactory clinical results of enhanced immune efficacy (114, 115). Additionally, multisite SBRT combined with ICIs was explored, achieved good efficacy, and even induced ABE (115, 116), providing an alternative for patients who cannot use ICIs.

Lastly, we emphasize that emerging evidence has revealed immunomodulatory capacities across both conventional and novel radiotherapy platforms—including comparative efficacy studies of different radiation modalities (e.g., photons versus particles, low-dose pulsed radiotherapy approaches. While these investigations fall beyond the scope of the current systematic review, we acknowledge this as a potential limitation and welcome further scholarly discourse to advance this evolving field.

## 5 Conclusion

In era of ICIs, the role of RT in regulating immunity remains unclear. Various RT methods aimed to stimulate the body’s anti-tumor immune response by engaging in different stages of immune responses, including the generating of local immune antigens, promoting the proliferation and activation of antigen-presenting cells and alter the immune environment and other links. In addition to SBRT and LDRT, some new RT techniques, such as FLASH-RT and carbon ion RT, have also been utilized. The impact of new techniques on the immune system and whether they can promote distant effects remain inconclusive; So dose some new combination of conventional radiotherapy techniques approaches are being actively explored in this field. We belive, with the rapid development of localized therapy and the widespread use of ICIs, more ABE-focused studies will be conducted, and RT, which has been shown to enhance systemic ICIs, is promising.
